# 1-(Morpholino­meth­yl)indoline-2,3-dione

**DOI:** 10.1107/S1600536810023160

**Published:** 2010-06-23

**Authors:** Ying Tang, Jie Zhang, Yanqing Miao, Gang Chen

**Affiliations:** a College of Chemistry and Chemical Engineering, Xi’an Shiyou University, Second Dianzi Road, Xi’an 710065, People’s Republic of China; b Department of Pharmacy, Xi’an Medical University, Hanguang Round 137, Xi’an 710021, People’s Republic of China

## Abstract

In the title compound, C_13_H_14_N_2_O_3_, the morpholine ring displays a chair conformation, with the (2,3-dioxoindolin-1-yl)methyl group in an equatorial position. The crystal structure is stabilized by inter­molecular C—H⋯O hydrogen bonds.

## Related literature

For the synthesis of isatin-*N*-Mannich bases, see: Varma & Nobles ((1966 ▶). For the bioactivity of isatin derivatives, see: Glover *et al.* (1980 ▶, 1988 ▶); Maysinger *et al.* (1980 ▶).
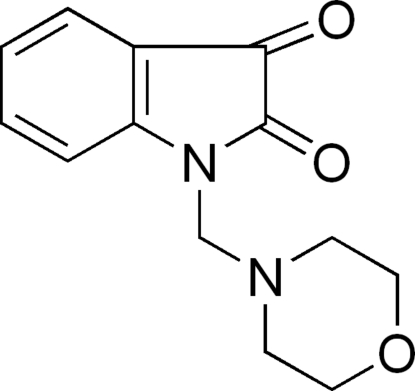


## Experimental

### 

#### Crystal data

C_13_H_14_N_2_O_3_
*M* *_r_* = 246.26
Monoclinic, 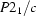

*a* = 11.608 (2) Å
*b* = 8.2818 (17) Å
*c* = 12.595 (3) Å
β = 100.20 (3)°
*V* = 1191.7 (4) Å^3^
*Z* = 4
Mo *K*α radiation
μ = 0.10 mm^−1^
*T* = 293 K
0.26 × 0.18 × 0.16 mm

#### Data collection

Bruker SMART CCD diffractometer
Absorption correction: multi-scan (*SADABS*; Bruker, 2001 ▶) *T*_min_ = 0.979, *T*_max_ = 0.984
7614 measured reflections
3556 independent reflections
2275 reflections with *I* > 2σ(*I*)
*R*_int_ = 0.021

#### Refinement

*R*[*F* ^2^ > 2σ(*F* ^2^)] = 0.042
*wR*(*F* ^2^) = 0.144
*S* = 0.99
3556 reflections
171 parameters
H atoms treated by a mixture of independent and constrained refinement
Δρ_max_ = 0.19 e Å^−3^
Δρ_min_ = −0.22 e Å^−3^

### 

Data collection: *SMART* (Bruker, 2001 ▶); cell refinement: *SAINT-Plus* (Bruker, 2001 ▶); data reduction: *SAINT-Plus*; program(s) used to solve structure: *SHELXS97* (Sheldrick, 2008 ▶); program(s) used to refine structure: *SHELXL97* (Sheldrick, 2008 ▶); molecular graphics: *SHELXTL* (Sheldrick, 2008 ▶); software used to prepare material for publication: *SHELXTL*.

## Supplementary Material

Crystal structure: contains datablocks I, global. DOI: 10.1107/S1600536810023160/rz2460sup1.cif

Structure factors: contains datablocks I. DOI: 10.1107/S1600536810023160/rz2460Isup2.hkl

Additional supplementary materials:  crystallographic information; 3D view; checkCIF report

## Figures and Tables

**Table 1:** Hydrogen-bond geometry (Å, °)

*D*—H⋯*A*	*D*—H	H⋯*A*	*D*⋯*A*	*D*—H⋯*A*
C6—H6*A*⋯O1^i^	0.93	2.47	3.349 (2)	158
C5—H5*A*⋯O2^ii^	0.93	2.52	3.216 (2)	131

Symmetry codes: (i) 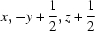; (ii) 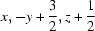.

**Fig. 1.**
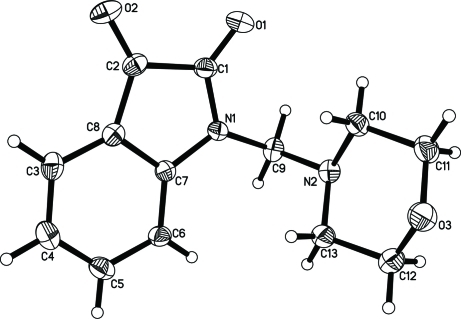
An ORTEP-3 drawing of the title compound, with the atom-numbering scheme and 30% probability displacement ellipsoids.

## supplementary crystallographic information

### Comment

Isatin has draw great attention from being discovered as a component of endogenous monoamine oxidase (MAO) inhibitory activity  (tribulin) and subsequently identified as a selective inhibitor of MAO B at low concentrations (Glover *et al.*, 1980, 1988). In the following years, many isatin derivatives, such as isatin hydrazono, isatin Mannich bases and isatin based spiroazetidinones, have also been reported to possess anticonvulsant activity (Maysinger, *et al.*, 1980). Herein we report the synthesis and crystal structure of the title compound.

The X-ray structural analysis confirmed the assignment of the structure from spectroscopic data. The molecular structure of the title compound is depicted in Fig. 1. Geometric parameters of the title compound are in the usual ranges. The morpholin ring displays a typical chair conformation, with the (2,3-dioxoindolin-1-yl)methyl group in equatorial position. In the crystal structure, the molecules are linked into a three-dimensional network by C—H···O hydrogen bonds (Table 1).

### Experimental

The title compound was synthesized according the literature method (Varma & Nobles, 1966). Isatin (1 mmol), formaldehyde (1.2 mmol) and morpholin (1.2 mmol) were dissolved in methanol (20 ml). The mixture was refluxed until the disappearance of isatin, as evidenced by thin-layer chromatography. The solvent was removed *in vacuo* and the residue was separated by column chromatography (silica gel, petroleum ether/ethyl acetate = 1:1 *v*/*v*), giving the title compound. ^1^H-NMR (D_6_—DMSO, 400 MHz): 7.61 (2*H*, m), 7.15 (1*H*, t, J = 7.2 Hz), 7.09 (1*H*, d, J = 8.0 Hz), 4.45 (2*H*, s), 3.70 (4*H*, t, J = 4.8 Hz), 2.63 (4*H*, t, J = 4.8 Hz); MS (EI) m/*z*: 246 (*M*^+^). 20 mg of the title compound was dissolved in 50 ml methanol and the solution was kept at room temperature for 4 d. Slow evaporation of the solvent gave orange single crystals of the title compound suitable for X-ray analysis.

### Refinement

The H atoms attached to atom C9 were located in a difference Fourier map and refined freely. All other H atoms were placed at calculated positions and refined as riding, with C—H = 0.93-0.98 Å, and with *U*_iso_(H) = 1.2*U*_eq_(C).

### Crystal data

**Table:**

C_13_H_14_N_2_O_3_	*F*(000) = 520
*M**_r_* = 246.26	*D*_x_ = 1.373 Mg m^−^^3^
Monoclinic, *P*2_1_/*c*	Mo *K*α radiation, λ = 0.71073 Å
Hall symbol: -P 2ybc	Cell parameters from 7356 reflections
*a* = 11.608 (2) Å	θ = 1.5–25.0°
*b* = 8.2818 (17) Å	µ = 0.10 mm^−^^1^
*c* = 12.595 (3) Å	*T* = 293 K
β = 100.20 (3)°	Block, orange
*V* = 1191.7 (4) Å^3^	0.26 × 0.18 × 0.16 mm
*Z* = 4	

### Data collection

**Table:**

Bruker SMART CCD diffractometer	3556 independent reflections
Radiation source: fine-focus sealed tube	2275 reflections with *I* > 2σ(*I*)
graphite	*R*_int_ = 0.021
phi and ω scans	θ_max_ = 30.4°, θ_min_ = 1.8°
Absorption correction: multi-scan (*SADABS*; Bruker, 2001)	*h* = −15→15
*T*_min_ = 0.979, *T*_max_ = 0.984	*k* = −11→10
7614 measured reflections	*l* = −17→15

### Refinement

**Table:**

Refinement on *F*^2^	Primary atom site location: structure-invariant direct methods
Least-squares matrix: full	Secondary atom site location: difference Fourier map
*R*[*F*^2^ > 2σ(*F*^2^)] = 0.042	Hydrogen site location: inferred from neighbouring sites
*wR*(*F*^2^) = 0.144	H atoms treated by a mixture of independent and constrained refinement
*S* = 0.99	*w* = 1/[σ^2^(*F*_o_^2^) + (0.1*P*)^2^] where *P* = (*F*_o_^2^ + 2*F*_c_^2^)/3
3556 reflections	(Δ/σ)_max_ = 0.039
171 parameters	Δρ_max_ = 0.19 e Å^−^^3^
0 restraints	Δρ_min_ = −0.22 e Å^−^^3^

### Special details

**Table:**

Geometry. All e.s.d.'s (except the e.s.d. in the dihedral angle between two l.s. planes) are estimated using the full covariance matrix. The cell e.s.d.'s are taken into account individually in the estimation of e.s.d.'s in distances, angles and torsion angles; correlations between e.s.d.'s in cell parameters are only used when they are defined by crystal symmetry. An approximate (isotropic) treatment of cell e.s.d.'s is used for estimating e.s.d.'s involving l.s. planes.
Refinement. Refinement of *F*^2^ against ALL reflections. The weighted *R*-factor *wR* and goodness of fit *S* are based on *F*^2^, conventional *R*-factors *R* are based on *F*, with *F* set to zero for negative *F*^2^. The threshold expression of *F*^2^ > σ(*F*^2^) is used only for calculating *R*-factors(gt) *etc*. and is not relevant to the choice of reflections for refinement. *R*-factors based on *F*^2^ are statistically about twice as large as those based on *F*, and *R*- factors based on ALL data will be even larger.

### Fractional atomic coordinates and isotropic or equivalent isotropic displacement parameters (Å^2^)

**Table:**

	*x*	*y*	*z*	*U*_iso_*/*U*_eq_	
N2	0.91766 (9)	0.23385 (12)	0.11391 (8)	0.0368 (3)	
N1	0.73127 (8)	0.33226 (11)	0.00535 (8)	0.0347 (3)	
C7	0.68098 (9)	0.48053 (14)	0.03062 (9)	0.0313 (3)	
O2	0.59676 (8)	0.46330 (13)	−0.24949 (7)	0.0527 (3)	
C10	0.98088 (11)	0.23531 (16)	0.02322 (11)	0.0414 (3)	
H10A	0.9591	0.3302	−0.0209	0.050*	
H10B	0.9604	0.1404	−0.0212	0.050*	
O3	1.14402 (7)	0.37200 (13)	0.13337 (9)	0.0564 (3)	
O1	0.73752 (10)	0.19019 (12)	−0.15122 (8)	0.0567 (3)	
C6	0.68346 (10)	0.54811 (15)	0.13085 (10)	0.0379 (3)	
H6A	0.7192	0.4957	0.1934	0.046*	
C8	0.62332 (10)	0.55651 (14)	−0.06320 (9)	0.0345 (3)	
C2	0.63447 (10)	0.45189 (15)	−0.15409 (10)	0.0377 (3)	
C9	0.79252 (11)	0.21364 (15)	0.08256 (11)	0.0373 (3)	
C5	0.63034 (11)	0.69807 (15)	0.13481 (11)	0.0456 (3)	
H5A	0.6323	0.7477	0.2014	0.055*	
C1	0.70789 (11)	0.30626 (15)	−0.10310 (10)	0.0385 (3)	
C3	0.56961 (11)	0.70484 (16)	−0.05764 (11)	0.0444 (3)	
H3A	0.5311	0.7557	−0.1197	0.053*	
C13	0.95237 (11)	0.37262 (17)	0.18371 (11)	0.0435 (3)	
H13A	0.9126	0.3692	0.2452	0.052*	
H13B	0.9303	0.4718	0.1443	0.052*	
C4	0.57469 (12)	0.77531 (16)	0.04263 (12)	0.0495 (4)	
H4A	0.5403	0.8757	0.0481	0.059*	
C12	1.08328 (12)	0.3691 (2)	0.22195 (12)	0.0540 (4)	
H12A	1.1065	0.4616	0.2680	0.065*	
H12B	1.1043	0.2721	0.2642	0.065*	
C11	1.11103 (11)	0.23690 (17)	0.06581 (12)	0.0514 (4)	
H11A	1.1329	0.1385	0.1062	0.062*	
H11B	1.1529	0.2397	0.0057	0.062*	
H9A	0.7745 (10)	0.1067 (17)	0.0422 (11)	0.038 (3)*	
H9B	0.7566 (11)	0.2191 (14)	0.1471 (11)	0.036 (3)*	

### Atomic displacement parameters (Å^2^)

**Table:**

	*U*^11^	*U*^22^	*U*^33^	*U*^12^	*U*^13^	*U*^23^
N2	0.0360 (5)	0.0363 (5)	0.0385 (6)	0.0006 (4)	0.0077 (4)	0.0042 (4)
N1	0.0366 (5)	0.0373 (5)	0.0306 (5)	0.0046 (4)	0.0074 (4)	−0.0023 (4)
C7	0.0285 (5)	0.0345 (6)	0.0319 (6)	−0.0018 (4)	0.0082 (4)	−0.0003 (4)
O2	0.0613 (6)	0.0656 (7)	0.0290 (5)	−0.0100 (5)	0.0018 (4)	0.0040 (4)
C10	0.0420 (7)	0.0404 (7)	0.0440 (7)	0.0003 (5)	0.0135 (6)	−0.0044 (5)
O3	0.0414 (5)	0.0612 (6)	0.0685 (7)	−0.0114 (4)	0.0150 (5)	−0.0142 (5)
O1	0.0671 (7)	0.0587 (6)	0.0452 (6)	0.0075 (5)	0.0125 (5)	−0.0178 (5)
C6	0.0385 (6)	0.0456 (7)	0.0302 (6)	0.0014 (5)	0.0073 (5)	−0.0019 (5)
C8	0.0316 (6)	0.0402 (6)	0.0322 (6)	−0.0026 (5)	0.0072 (5)	0.0032 (5)
C2	0.0352 (6)	0.0475 (7)	0.0304 (6)	−0.0072 (5)	0.0058 (5)	0.0025 (5)
C9	0.0361 (6)	0.0362 (6)	0.0403 (7)	−0.0010 (5)	0.0086 (5)	0.0055 (5)
C5	0.0474 (8)	0.0460 (7)	0.0458 (8)	0.0010 (6)	0.0146 (6)	−0.0114 (6)
C1	0.0384 (6)	0.0449 (7)	0.0331 (6)	−0.0028 (5)	0.0089 (5)	−0.0053 (5)
C3	0.0423 (7)	0.0432 (7)	0.0474 (8)	0.0050 (5)	0.0074 (6)	0.0115 (6)
C13	0.0416 (7)	0.0487 (7)	0.0409 (7)	0.0013 (6)	0.0092 (6)	−0.0057 (6)
C4	0.0488 (8)	0.0391 (7)	0.0623 (9)	0.0076 (6)	0.0145 (7)	−0.0014 (6)
C12	0.0440 (7)	0.0652 (9)	0.0509 (9)	−0.0030 (7)	0.0033 (7)	−0.0131 (7)
C11	0.0405 (7)	0.0549 (8)	0.0611 (9)	−0.0008 (6)	0.0157 (7)	−0.0116 (7)

### Geometric parameters (Å, °)

**Table:**

N2—C9	1.4460 (16)	C8—C3	1.3850 (17)
N2—C13	1.4596 (16)	C8—C2	1.4597 (17)
N2—C10	1.4631 (16)	C2—C1	1.5490 (18)
N1—C1	1.3618 (15)	C9—H9A	1.024 (14)
N1—C7	1.4199 (14)	C9—H9B	0.979 (14)
N1—C9	1.4736 (15)	C5—C4	1.382 (2)
C7—C6	1.3765 (16)	C5—H5A	0.9300
C7—C8	1.4000 (15)	C3—C4	1.383 (2)
O2—C2	1.2075 (15)	C3—H3A	0.9300
C10—C11	1.5110 (18)	C13—C12	1.5109 (18)
C10—H10A	0.9700	C13—H13A	0.9700
C10—H10B	0.9700	C13—H13B	0.9700
O3—C11	1.4168 (16)	C4—H4A	0.9300
O3—C12	1.4217 (17)	C12—H12A	0.9700
O1—C1	1.2180 (14)	C12—H12B	0.9700
C6—C5	1.3914 (17)	C11—H11A	0.9700
C6—H6A	0.9300	C11—H11B	0.9700
			
C9—N2—C13	114.36 (10)	C4—C5—C6	121.82 (12)
C9—N2—C10	113.97 (10)	C4—C5—H5A	119.1
C13—N2—C10	109.92 (10)	C6—C5—H5A	119.1
C1—N1—C7	110.17 (10)	O1—C1—N1	126.89 (12)
C1—N1—C9	122.94 (10)	O1—C1—C2	126.25 (12)
C7—N1—C9	126.73 (10)	N1—C1—C2	106.86 (10)
C6—C7—C8	121.37 (11)	C4—C3—C8	118.27 (12)
C6—C7—N1	127.92 (11)	C4—C3—H3A	120.9
C8—C7—N1	110.71 (10)	C8—C3—H3A	120.9
N2—C10—C11	109.34 (11)	N2—C13—C12	109.35 (11)
N2—C10—H10A	109.8	N2—C13—H13A	109.8
C11—C10—H10A	109.8	C12—C13—H13A	109.8
N2—C10—H10B	109.8	N2—C13—H13B	109.8
C11—C10—H10B	109.8	C12—C13—H13B	109.8
H10A—C10—H10B	108.3	H13A—C13—H13B	108.3
C11—O3—C12	109.81 (11)	C3—C4—C5	120.66 (13)
C7—C6—C5	117.28 (11)	C3—C4—H4A	119.7
C7—C6—H6A	121.4	C5—C4—H4A	119.7
C5—C6—H6A	121.4	O3—C12—C13	111.11 (12)
C3—C8—C7	120.55 (11)	O3—C12—H12A	109.4
C3—C8—C2	132.02 (11)	C13—C12—H12A	109.4
C7—C8—C2	107.44 (10)	O3—C12—H12B	109.4
O2—C2—C8	131.85 (13)	C13—C12—H12B	109.4
O2—C2—C1	123.32 (12)	H12A—C12—H12B	108.0
C8—C2—C1	104.82 (10)	O3—C11—C10	111.53 (11)
N2—C9—N1	116.55 (10)	O3—C11—H11A	109.3
N2—C9—H9A	110.1 (7)	C10—C11—H11A	109.3
N1—C9—H9A	102.5 (7)	O3—C11—H11B	109.3
N2—C9—H9B	108.9 (7)	C10—C11—H11B	109.3
N1—C9—H9B	106.9 (7)	H11A—C11—H11B	108.0
H9A—C9—H9B	111.9 (10)		

### Hydrogen-bond geometry (Å, °)

**Table:**

*D*—H···*A*	*D*—H	H···*A*	*D*···*A*	*D*—H···*A*
C6—H6A···O1^i^	0.93	2.47	3.349 (2)	158
C5—H5A···O2^ii^	0.93	2.52	3.216 (2)	131

Symmetry codes: (i) *x*, −*y*+1/2, *z*+1/2; (ii) *x*, −*y*+3/2, *z*+1/2.
